# Maternal Age and Stage of Pregnancy as Determinants of UTI in Pregnancy: A Case of Tamale, Ghana

**DOI:** 10.1155/2022/3616028

**Published:** 2022-04-12

**Authors:** Jacob Loonin Laari, Martin Anab, Damyetin Peter Jabong, Kasim Abdulai, Abdul Rauf Alhassan

**Affiliations:** ^1^School of Hygiene Tamale, P.O. Box 88, Tamale, Ghana; ^2^College of Health Sciences Yendi, Ministry of Health, Ghana; ^3^Department of Clinical Nutrition and Dietetics, School of Allied Health Sciences, University of Cape Coast, Ghana; ^4^Department of Surgery, Tamale Teaching Hospital, P.O. Box TL 16, Tamale, Ghana

## Abstract

**Introduction:**

Urinary tract infection (UTI) is the world's second most common cause of death, trailing only respiratory tract infections. Because of anatomical and physiological changes along the urinary tract, pregnant women accounted for approximately 20% of all cases of urinary tract infection.

**Aim:**

This study sought to assess maternal age and stage of pregnancy as determinants of UTI among pregnant women in Tamale.

**Methods:**

This study employed a descriptive cross-sectional survey as the study design in the antenatal clinic of Tamale Central Hospital (TCH). This was carried out by reviewing laboratory records of urinalysis results done on pregnant women. Data entry and analysis were performed by the Statistical Package for the Social Sciences (SPSS) version 20. Chi-square and binary logistics analysis were used to determine the relationship.

**Results:**

Data analysis was done for 158 pregnant women, most (35.4%) were within the age group of 36-45 years, and most (38.6%) were within their first trimester. The overall prevalence of UTI infections among pregnant women was 33.5%. The prevalence was 27.8% for candiduria and 8.9% for bacteriuria. Women in the first trimester of their pregnancy were more likely to have UTI (AOR = 2.48, 95% CI =1.03–5.94). Also, Pregnant women of the age group of 26-35 years were less likely to get UTIs as compared to those of the age group 15-25 years (AOR = 0.40, 95% CI =0.17–0.92). Finally, those of the age group of 36-45 years were less likely to get UTI as compared to those of the age group 15-25 years (AOR = 0.28, 95% CI =0.12–0.66).

**Conclusion:**

The prevalence of UTI among studied pregnant women was high (38.0%), and the most prone maternal age group and trimesters to UTI are 15-25 years and first trimester, respectively.

## 1. Introduction

Urinary tract infection (UTI) is the world's second most common cause of death, trailing only respiratory tract infections. UTIs are infections of the urinary tract, which include the urethra, urinary bladder, ureter, and kidney. Excrement-contaminated bacteria entering the urinary tract typically cause UTIs [1]. Since women have a shorter urethra than men do, they are more likely to suffer from UTIs. Because of anatomical and physiological changes along the urinary tract, pregnant women are at risk of urinary tract infection. Pregnant women accounted for approximately 20% of all cases of urinary tract infection [2–5].

Pregnant women are at increased risk of UTIs. UTIs affect nearly 2%–10% of pregnant women [6, 7]. Their diminished immunity appears to promote the growth of both commensal and non-commensal microorganisms [8]. Asymptomatic bacteriuria is a common symptom of UTI in pregnant women [7, 9, 10]. Asymptomatic bacteriuria in pregnancy is dangerous because untreated cases have a high likelihood (up to 40%) of progressing to acute pyelonephritis, which can induce morbidity and even death to the mother and fetus [9]. To avoid perinatal complications such as bacteremia, premature birth, and low birth weight, treatment must be initiated immediately [11, 12].

Anatomical and physiological changes, as well as the hormonal effects of pregnancy, all contribute to an increased risk of urinary tract infection. The effect of progesterone on muscle tone and motility, as well as mechanical obstruction caused by uterine enlargement, will dilate the pelvis calyces system and ureter, increasing urinary bladder capacity, vesicoureteral reflux, and rest urine after voiding, and, finally, an increased risk of urinary tract infection. Fetal head pressure also prevents blood and lymph drainage from the base of the urinary bladder, causing edema and vulnerability in the affected area. Furthermore, because women have a shorter urethra than men do, germs can more easily invade the perianal area, vagina, and rectum, increasing the risk of infection. Furthermore, the shorter urethra in women compared to men anatomically allows germs to more easily invade the perianal area, vagina, and rectum, increasing the risk of urinary tract infection in pregnant women [2–5, 13].

“*Escherichia coli*, *Staphylococcus* spp., *Streptococcus* spp., *Proteus* spp., *Klebsiella* spp., *Corynebacterium*, *Neisseria*, and *Pseudomonas* spp.” are the most commonly detected causes of urinary tract infection. The virulence of the *bacteria* and the host's susceptibility determines the intensity of urinary tract infections [14]. In another study, given the high prevalence of Trichomonas vaginalis, the authors recommend that women with recurrent UTIs undergo Trichomonas vaginalis testing. They further recommended more large-scale studies to assess the potential benefit of treating Trichomonas vaginalis in this patient population [15]. *Candida* species cause 12–17 percent of UTIs in hospitalized patients, with intensive care unit (ICU) patients being the most vulnerable [16]. The importance of a candiduria episode, on the other hand, is unknown. Although the majority of patients is asymptomatic, the presence of *Candida* in urine may indicate contamination, colonization, or even an infection that leads to pyelonephritis, fungus ball, and candidemia [17, 18].

Diabetes mellitus (prevalence of about 8-14 percent) and fecal incontinence are common risk factors for UTI. However, uropathogenic exposure, increasing factors of uropathogenic colonization, and immune response to uropathogenic colonization are all associated with UTI cases in pregnancy. Bacteriuria is more likely to occur between the 9th and 17th weeks of pregnancy. Asymptomatic bacteriuria affects 80% of pregnant women between the 12th and 16th weeks of their pregnancy. The highest incidence of UTI is at the 30th and 32nd weeks of pregnancy [2, 3, 5, 13]. This study sought to assess maternal age and stage of pregnancy as determinants of UTI among pregnant women in Tamale.

## 2. Methodology

### 2.1. Study Design and Site

This study employed a descriptive cross-sectional survey as the study design using a quantitative approach. The study took place in the antenatal clinic of Tamale Central Hospital (TCH). This was carried out by reviewing laboratory records of urinalysis results done on pregnant women.

### 2.2. Study Participants

In this current study, the population consists of pregnant women who visit TCH's antenatal clinic. According to the records of TCH's antenatal clinic, an average of 50 pregnant women attend the clinic daily. In addition, depending on the gestational weeks of the pregnancy, pregnant women must wait at least one week between visits. As a result, the population was determined by calculating for a five-working-day period to avoid subject duplication in the total population, and the total population for this study was 50 × 50 = 250 pregnant women.

### 2.3. Sampling and Sample Size Determination

The sample size for this study was determined using the Yamane formula for calculating sample size, which is below, where *n* is the sample size, *N* is the population size, and *e* is the level of precision. With the known population of 250 and level of precision set at 0.05, the formula can be calculated as

(1) $$\begin{array}{c} n=\frac{N}{1+N{\left(e\right)}^{2}}\;n=\frac{250}{1+250\;{\left(0.05\right)}^{2}}=153 \end{array}$$

Hence, a minimum of 153 participants were needed for this study. Systematic sampling technique with sampling interval 2 is applied for participants' selection from the laboratory record book for urinalysis of pregnant women. The first participant selection was by simple random sampling techniques selection.

### 2.4. Study Variables

The dependent variable in this study was urinary tract infection, presence of yeast in urine (candiduria), presence of *bacteria* (bacteriuria), or presence of both yeast and *bacteria*. In addition, the independent variables were maternal age and stage of pregnancy.

### 2.5. Method of Data Analysis

Data entry and analysis were performed by the Statistical Package for the Social Sciences (SPSS) version 20. Categorical variables were coded to allow for quantitative analysis. Data cleaning was performed to ensure data accuracy and maintain the study's good validity. Graphs and tables were used to present the study data. Chi-square and binary logistics analysis were used to determine the relationship.

### 2.6. Ethical Consideration

An application containing a summary study proposal and data collection protocol was submitted to TCH management for permission to conduct the research. Permission to conduct this study in the hospital was granted. Ethical approval for this study was not very required as secondary data was used. To avoid plagiarism, all sources for material used in this study are properly credited.

## 3. Results

### 3.1. Demographic Characteristics of Study Participants

Data analysis was done for 158 study pregnant women, most (35.4%) were within the age group of 36-45 years, 32.9% of them were within 26-35 years, and 31.6% were of the age group 15-25 years. In terms of the trimester stage of pregnancy, most (38.6%) were within their first trimester, 31.0% of them were within their second trimester, and the remaining 30.4% of them were within their third trimester of pregnancy (Table 1).

### 3.2. UTI among Pregnant Women

#### 3.2.1. Prevalence of UTI Infection with the under Four Studies Microorganisms

The microscopic urinalysis for the pregnant women revealed the following: trichomonas vaginalis (3.8%), yeast-like cells (27.8%), crystal (5.7%), and *bacteria* (8.9%). The overall prevalence of UTI infections among pregnant women was 33.5%. The prevalence was 27.8% for candiduria and 8.9% for bacteriuria (Table 2).

#### 3.2.2. Most Maternal Age and Trimester Prone to UTI Infection

Chi-square analysis did reveal a significant association between maternal age and UTI infection. The presence of UTI infection was proportionally high (50.0%) among pregnant women within the age group of 15-25 years (*X*^2^ = 9.269, *P* = 0.010) (Table 3). Proportionally, most (40.0%) of the candiduria infection were among those of the age group 15-25 years. In addition, bacteriuria infections were higher (16.0%) among those of the age group 15-25 years (Table 4).

In addition, at the two-variable analysis stage, the trimester of pregnancy did not make much difference in UTI infection with chi-square analysis (*X*^2^ = 3.975, *P* = 0.137). This is worth noting that proportionally UTI was higher (41.0%) in the first trimester of pregnancy (Table 3). Proportionally, candiduria was higher (30.6%) among those in the second trimester of their pregnancy. In addition, bacteriuria was higher (13.1%) among those in the first trimester of their pregnancy (Table 4).

#### 3.2.3. Maternal Age and Trimester of Pregnancy as a Predictor of UTI Infection in Pregnant Women

Further analysis from two variables stage using a binary logistics regression model to control for the age group of the study participants revealed the first trimester of predicted UTI in pregnant women. Women in the first trimester of their pregnancy were 148.0% more likely to get UTI infection as compared to their counterparts in their third trimester of pregnancy (AOR = 2.48, 95% CI =1.03–5.94). Also, the age group of pregnant women did have a prediction for UTI infection. Pregnant women of the age group of 26-35 years were 60.0% less likely to get UTI as compared to those of age group 15-25 years (AOR = 0.40, 95% CI =0.17–0.92). Also, pregnant women of the age group of 36-45 years were 72.0% less likely to get UTI as compared to those of the age group 15-25 years (AOR = 0.28, 95% CI =0.12–0.66) (Table 5).

## 4. Discussion

Pregnant women are at increased risk of UTIs. UTIs affect nearly 2%–10% of pregnant women [6, 7]. Pregnant women accounted for approximately 20% of all cases of urinary tract infection [2–5]. In this present study, the overall prevalence of UTI infections among pregnant women was 33.5%. The prevalence of this study is lower as compared to that of Forson et al.'s study which reported 42.8% and that of Boye et al., which also reported a prevalence of 56.5% which were both Ghanaian studies [19, 20].

Asymptomatic bacteriuria in pregnancy is dangerous because untreated cases have a high likelihood (up to 40%) of progressing to acute pyelonephritis, which can induce morbidity and even death to the mother and fetus [9]. The prevalence of bacteriuria reported in this study was 8.9% which is higher than earlier Ghanaian studies, 5.5% in Korle-bu Teaching Hospital, and 7.3% in Komfo-Anokye Teaching Hospital [21, 22]. The importance of a candiduria episode, on the other hand, is unknown. Although the majority of patients are asymptomatic, the presence of *Candida* in urine may indicate contamination, colonization, or even an infection that leads to pyelonephritis, fungus ball, and candidemia [17, 18]. The prevalence was 27.8% for candiduria in this present study. A ten-year retrospective study for inpatients and outpatients did reveal the prevalence of 65.2% for outpatients and 59.6% for inpatients [23].

In another study, given the high prevalence of Trichomonas vaginalis, the authors recommend that women with recurrent UTIs undergo Trichomonas vaginalis testing. They further recommended more large-scale studies to assess the potential benefit of treating Trichomonas vaginalis in this patient population [15]. This is not ignored in this present study; the microscopic urinalysis revealed trichomonas vaginalis organism in 3.8% of the pregnant women.

The age of 20 years is a risk factor for complicated UTI in pregnancy [24]. In this current study, the prevalence of UTI infection was proportionally high among pregnant women within the age group of 15-25 years. Pregnant women of the age group of 26-35 years were less likely to get UTIs as compared to those of the age group 15-25 years. In addition, pregnant women of the age group of 36-45 years were less likely to get UTIs as compared to those of the age group 15-25 years. Another study conducted by Fred et al. found a high percentage of UTIs in the age group 15-25 years [25]. However, in a study by Kerure et al., UTI prevalence was higher among those of the age group 26-35 years [26]. Proportionally, most of the candiduria infections were among those of the age group 15-25 years. In addition, bacteriuria infections were higher among those of the age group 15-25 years. Low socioeconomic status, young age, and null parity are all risk factors for UTIs in pregnancy [27]. Younger pregnant women are exposed to nutrition deficiency due to low economic status and lack of knowledge on nutritional requirements [28]. Pregnancy is already a risk factor for UTI, and then, poor nutrition in pregnancy will be the greater risk.

This is worth noting that proportionally UTI was higher in the first trimester of pregnancy. Proportionally, candiduria was higher among those in the second trimester of their pregnancy. In addition, bacteriuria was higher among those in the first trimester of their pregnancy. Other previous studies have it that bacteriuria is more likely to occur between the 9th and 17th weeks of pregnancy [2, 3, 5, 13]. This current study is further analyzed by using a binary logistics regression model to control for the age group of the study participants which revealed the first trimester of predicted UTI in pregnant women. Women in the first trimester of their pregnancy were more likely to get UTI infections as compared to their counterparts in their third trimester of pregnancy. Approximately, 41% of UTIs are diagnosed during the first trimester, since UTIs are so common during the first trimester [29]. Hormone production is a major risk for UTI infection [2]. Rapid changes during pregnancy and hormonal production are associated with the first trimester of pregnancy, and it is at this stage of pregnancy that women suffer unpleasant pregnancy symptoms.

## 5. Conclusion

In conclusion, the prevalence of UTI among studied pregnant women was high (38.0%), and the most prone maternal age group and trimester to UTI is 15-25 years and first trimester, respectively.

## 6. Recommendation

A future study using primary data to the evaluated economic and nutritional status of the expectant mother as predictor factors of UTI in pregnancy in the study area is recommended.

## Figures and Tables

**Table 1:** Demographic characteristics of study participants.

		Frequency	Percent
Age group	15-25 years	50	31.6
	26-35 years	52	32.9
	36-45 years	56	35.4
	Total	158	100.0
Trimester	First	61	38.6
	Second	49	31.0
	Third	48	30.4
	Total	158	100.0

Source: Field data (2021).

**Table 2:** Results of urinalysis and prevalence of UTI among the pregnant women.

		Frequency	Percent
*Results of urinalysis for the pregnant women*			
Trichomonas vaginalis	Absent	152	96.2
	Present	6	3.8
	Total	158	100.0
Yeast-like cell	Absent	114	72.2
	Present	44	27.8
	Total	158	100.0
Crystals	Absent	149	94.3
	Present	9	5.7
	Total	158	100.0
*Bacteria*	Absent	144	91.1
	Present	14	8.9
	Total	158	100.0
*Prevalence of UTI infections among pregnant women*			
Candiduria	No	114	72.2
	Yes	44	27.8
Bacteriuria	Total	158	100.0
	No	144	91.1
	Yes	14	8.9
	Total	158	100.0
Overall UTI infection	No	105	66.5
	Yes	53	33.5
	Total	158	100.0

Source: Field data (2021).

**Table 3:** Association between maternal age, trimester of pregnancy, and UTI infection.

		UTI infection		Test statistics
		No	Yes	
Age group	15-25 years	25	25	*X* ^2^ = 9.269 *P* = 0.010
		50.0%	50.0%	
	26-35 years	37	15	
		71.2%	28.8%	
	36-45 years	43	13	
		76.8%	23.2%	
Total		105	53	
		66.5%	33.5%	
Trimester	First	36	25	*X* ^2^ = 3.975 *P* = 0.137
		59.0%	41.0%	
	Second	32	17	
		65.3%	34.7%	
	Third	37	11	
		77.1%	22.9%	
Total		105	53	
		66.5%	33.5%	

Source: Field data (2021).

**Table 4:** Association between maternal age, stage of pregnancy, and identified microorganism.

		Candiduria		Total	Bacteriuria		Total
		No	Yes		No	Yes	
Age group	15-25 years	30	20	50	42	8	50
		60.0%	40.0%	100.0%	84.0%	16.0%	100.0%
	26-35 years	41	11	52	46	6	52
		78.8%	21.2%	100.0%	88.5%	11.5%	100.0%
	36-45 years	43	13	56	56	0	56
		76.8%	23.2%	100.0%	100.0%	0.0%	100.0%
Total		114	44	158	144	14	158
		72.2%	27.8%	100.0%	91.1%	8.9%	100.0%
Trimester	First	43	18	61	53	8	61
		70.5%	29.5%	100.0%	86.9%	13.1%	100.0%
	Second	34	15	49	43	6	49
		69.4%	30.6%	100.0%	87.8%	12.2%	100.0%
	Third	37	11	48	48	0	48
		77.1%	22.9%	100.0%	100.0%	0.0%	100.0%
Total		114	44	158	144	14	158
		72.2%	27.8%	100.0%	91.1%	8.9%	100.0%

Source: Field data (2021).

**Table 5:** Maternal age and trimester of pregnancy as a predictor of UTI infection in pregnant women.

		*B*	Wald	*P* value	AOR	95% CI for AOR	
						Lower	Upper
Age group	15-25 years		9.292	.010			
	26-35 years	-.913	4.617	*.032*	*.401*	*.174*	*.923*
	36-45 years	-1.261	8.457	*.004*	*.283*	*.121*	*.663*
Trimester	Third		4.265	.119			
	First	.908	4.143	*.042*	*2.479*	*1.034*	*5.943*
	Second	.710	2.256	.133	2.034	.805	5.139
	Constant	-.569	1.977	.160	.566		

Source: Field data (2021).

## Data Availability

All data relating to the findings of this study are available from the corresponding author on request.
